# A Simple Preparation Method of Gelatin Hydrogels Incorporating Cisplatin for Sustained Release

**DOI:** 10.3390/pharmaceutics14122601

**Published:** 2022-11-25

**Authors:** Takahisa Suzuki, Shigeru Tsunoda, Kota Yamashita, Toshie Kuwahara, Mitsuru Ando, Yasuhiko Tabata, Kazutaka Obama

**Affiliations:** 1 Department of Surgery, Graduate School of Medicine, Kyoto University, Kyoto 606-8501, Japan; 2 Laboratory of Biomaterials, Department of Regeneration Science and Engineering, Institute for Life and Medical Sciences, Kyoto University, Kyoto 606-8507, Japan

**Keywords:** gelatin hydrogel, sustained release, crosslinking method, cisplatin, gastric cancer, peritoneal metastases

## Abstract

The objective of this study was to develop a new preparation method for cisplatin (CDDP)-incorporated gelatin hydrogels without using chemical crosslinking nor a vacuum heating instrument for dehydrothermal crosslinking. By simply mixing CDDP and gelatin, CDDP-crosslinked gelatin hydrogels (CCGH) were prepared. CDDP functions as a crosslinking agent of gelatin to form the gelatin hydrogel. Simultaneously, CDDP is incorporated into the gelatin hydrogel as a controlled release carrier. CDDP’s *in vitro* and *in vivo* anticancer efficacy after incorporation into CCGH was evaluated. In the *in vitro* system, the CDDP was released gradually due to CCGH degradation with an initial burst release of approximately 16%. CDDP metal-coordinated with the degraded fragment of gelatin was released from CCGH with maintaining the anticancer activity. After intraperitoneal administration of CCGH, CDDP was detected in the blood circulation while its toxicity was low. Following intraperitoneal administration of CCGH in a murine peritoneal dissemination model of human gastric cancer MKN45-Luc cell line, the survival time was significantly prolonged compared with free CDDP solution. It is concluded that CCGH prepared by the CDDP-based crosslinking of gelatin is an excellent sustained release system of CDDP to achieve superior anticancer effects with minimal side effects compared with free CDDP solution.

## 1. Introduction

The burden of cancer incidence and mortality are rapidly growing worldwide, with an estimated 19.3 million new cancer cases and approximately 10 million cancer deaths in 2020 [1]. Despite rapid progress in diagnostics and therapeutics, cancer remains one of the most severe threats to humans [2]. Chemotherapy is the first-line treatment for metastatic cancer. However, the clinical efficacy of chemotherapy is limited by several pharmacological parameters associated with the anticancer drug, such as low aqueous solubility, short circulation half-life *in vivo*, lack of stability, and non-selective drug distribution resulting in alleviated therapeutic effect and severe side effects [2,3]. For example, Cisplatin (cis-diamminedichloroplatinum(II), CDDP) is one of the most effective anticancer drugs for various types of cancer. It has been used for a long time since its approval by the Food and Drug Administration (FDA) in 1978. However, its clinical applications are often limited because of severe side effects like nephrotoxicity, myelosuppression, nausea, and vomiting [4,5,6].

**Figure 1:**
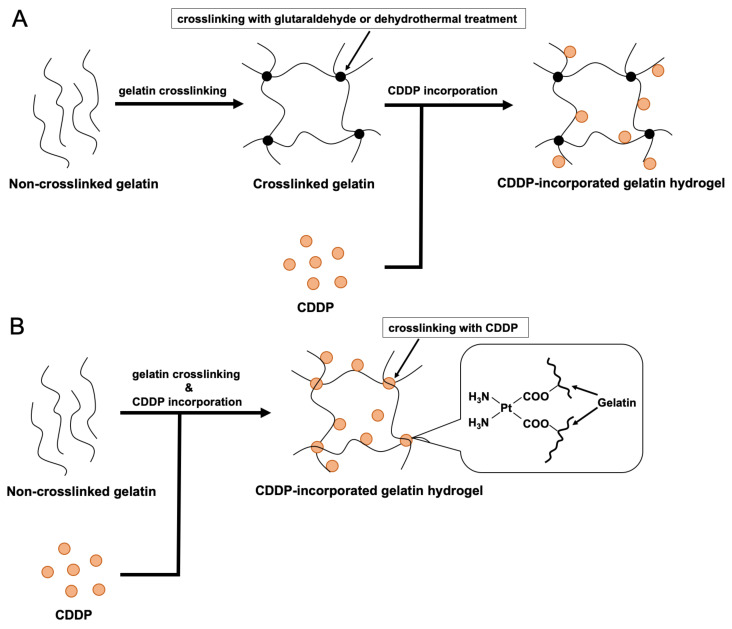
Preparation method for gelatin hydrogels incorporating CDDP for sustained CDDP release. (**A**) Two-step method reported previously. First, gelatin is crosslinked by chemical crosslinking using glutaraldehyde or dehydrothermal crosslinking, and then the CDDP is incorporated into it. (**B**) The newly developed one-step method. By crosslinking gelatin with CDDP, gelatin crosslinking and CDDP incorporation are performed simultaneously. The binding mechanism between CDDP and gelatin molecules can be a coordinate bond between platinum atoms and the carboxyl groups of gelatin side chains.

In recent years, many studies on new drug delivery systems (DDSs) have been carried out to overcome the shortcomings of chemotherapy, such as liposomes [7,8], polymeric micelles [9], mesoporous silica nanoparticles [10], dendrimers [11], and nanosuspensions [12,13,14]. We have focused on gelatin as a carrier for release-controlled DDS [15,16,17,18,19,20,21,22]. Gelatin, a denatured form of collagen, is an attractive biomaterial due to its biodegradability, high biocompatibility, biosafety, and cost-effectiveness. Gelatin hydrogels of different shapes have been experimentally demonstrated as a carrier for DDS, cell culture substrate [23,24,25], and scaffold for tissue regeneration [26,27,28,29,30,31,32,33]. Since gelatin dissolves rapidly in an aqueous environment, it should be crosslinked for use as a carrier for sustained drug release. The drug release from gelatin can be easily controlled by controlling the degree of crosslinking of gelatin molecules [15,34,35]. The primary crosslinking method is the chemical crosslinking using a crosslinking agent or dehydrothermal crosslinking. It has been demonstrated that the degree of crosslinking can be controlled by optimizing the dose of the chemical crosslinking agent, heating temperature, and heating time, respectively [36]. We have previously reported CDDP-incorporated gelatin hydrogels which utilized gelatin hydrogels as a carrier for the sustained release of CDDP to experimentally demonstrate a highly efficacious anticancer activity with negligible side effects [37,38,39,40]. The CDDP-incorporated gelatin hydrogels were prepared by a two-step method. In brief, hydrogels were prepared by crosslinking gelatin, and CDDP was incorporated into the hydrogels. Gelatin was chemically crosslinked with glutaraldehyde or dehydrothermal treatment (Figure 1A). However, each method has room to be improved. Glutaraldehyde is highly toxic and irritating. The latter requires a special instrument for vacuum and heating for a rather long time (two days).

In this study, we designed a novel preparation method for CDDP-incorporated gelatin hydrogels, which overcomes the drawbacks mentioned above. The anticancer drug CDDP was used as a gelatin crosslinking agent in this preparation process. Both gelatin crosslinking and CDDP incorporation into gelatin can be achieved simultaneously (Figure 1B). Herein, we examined the *in vitro* degradation and CDDP release profiles of CDDP-crosslinked gelatin hydrogels (CCGH) prepared by this one-step method. We examined the preparation condition of CCGH in terms of the CDDP loading in CCGH. Furthermore, we evaluated the *in vitro* anticancer efficacy. Following the intraperitoneal administration of CCGH into a mouse model of peritoneal cancer dissemination, we investigated the anticancer efficacy and the adverse effects.

## 2. Materials and Methods

### 2.1. Chemicals

Gelatin (beMatrix® LS-H) with a low level of endotoxin, an isoelectric point of 5.0, and a molecular weight of 100,000 prepared by an alkaline process using porcine skin was kindly supplied by Nitta Gelatin, Inc. (Osaka, Japan). CDDP was purchased from Wako Pure Chemical Industries, Ltd. (Osaka, Japan). Luciferin was purchased from Promega (Madison, WI, USA).

### 2.2. Cells

A poorly differentiated human gastric adenocarcinoma cell line expressing luciferase (MKN45-Luc) was purchased from the JGRB Cell Bank, National Institutes of Biomedical Innovation, Health and Nutrition (Osaka, Japan). The cells were cultured in RPMI-1640 (Thermo Fisher Scientific, Inc., Waltham, MA, USA) supplemented with 10% (vol) fetal bovine serum (Thermo Fisher Scientific, Inc.), 50 U/mL of penicillin (Nacalai Tesque, Inc, Kyoto, Japan) and 50 U/mL of streptomycin (Nacalai Tesque, Inc.) in a humidified atmosphere with 5% CO_2_ at 37 °C.

### 2.3. Animals

Six-week-old female BALB/c nude mice (16–20 g body weight) and five-week-old female ddY mice (22–24 g body weight) were purchased from Shimizu Laboratory Supplies Co, Ltd. (Kyoto, Japan). All animal procedures were performed by the Guidelines for the Care and Use of Laboratory Animals published by the National Institute of Health. The Institutional Animal Experiment Committee of Kyoto University approved this study protocol (No. F-21-247).

### 2.4. Preparation of CDDP-Crosslinked Gelatin Hydrogel Granules

CCGH granules were prepared using CDDP as a crosslinking agent. In brief, 1 g of gelatin was swelled in 10 mL double deionized water (DDW) at room temperature for 30 min and then dissolved at 40 °C for 1 h. Then, 30 mL CDDP aqueous solution (3 mg/mL) was added to the dissolved gelatin solution and stirred at 40 °C for 6 h. The mixed aqueous solution was cast into a polystyrene dish and left at 4 °C for 24 h. The resulting hydrogel sheets were smashed by putting through two sieves with apertures of 108 and 75 μm (Iida Seisakusho Co, Ltd., Osaka, Japan). The resulting hydrogel granules were stirred in ethanol, and the supernatant was removed after spontaneous sedimentation at 4 °C. This process was repeated five times for dehydration of the granules and removing uncombined CDDP from the granules. Finally, the granules were degassed in a desiccator overnight to remove ethanol and then sterilized with ethylene oxide gas.

### 2.5. Characterization of CCGH: In Vitro

Dried CCGH and CCGH dispersed in DDW were imaged using a microscope (BZ-X710, KEYENCE Ltd., Osaka, Japan). The CDDP content of CCGH was evaluated. Herein, 5 mg of CCGH was placed in 1 mL of 0.01 M phosphate-buffered saline (PBS) containing collagenase D (100 μg/mL) and was left at 37 °C for 24 h for complete dissolution. The CDDP concentration in the dissolved solution was measured based on platinum detected by a polarized Zeeman Z-8000 atomic absorption spectrophotometer (Hitachi, Ltd., Tokyo, Japan).

The degradability of CCGH and the CDDP release from CCGH *in vitro* were evaluated. Herein, 7.5 mg of CCGH was added to 1.5 mL PBS, followed by reciprocal shaking at 60 strokes/min at 37 °C. At pre-determined time intervals (1, 6, 12, 13, 15, 18, 24, and 30 h), 200 μL of the supernatant was collected after centrifugation (5000 rpm, 4 °C, 10 min) and replaced with the same volume of PBS. After 12 h, PBS was replaced with PBS containing collagenase D so that the concentration of collagenase in the solution was 10 μg/mL. The degradability of CCGH was assessed by quantitating proteins in the supernatant with Micro BCATM Protein Assay Kit (Thermo Fisher Scientific, Inc.), and the CDDP release from CCGH was assessed by quantitating platinum in the supernatant with a polarized Zeeman Z-8000 atomic absorption spectrophotometer.

### 2.6. Evaluation of CCGH Preparation Conditions

To evaluate the effect of the preparation conditions on CCGH crosslinking degree, CCGH was prepared by varying the preparation conditions. Specifically, we examined the stirring time (1, 3, 6, and 12 h) of the gelatin aqueous solution and the CDDP aqueous solution at 40 °C. We also examined the standing time (6, 12, 24, and 48 h) at 4 °C after stirring. CDDP content, degradability, and CDDP release from CCGH prepared under each condition were assessed by the above methods. In addition, we evaluated the recovery rate of the CCGH stirred for 6 h by varying the standing time (6, 12, 24, and 48 h) at 4 ℃. The recovery rate was calculated as the percentage of the weight of the prepared CCGH relative to the total weight of gelatin and CDDP used for the preparation.

### 2.7. Evaluation of In Vitro Cytotoxicity of CCGH

We assessed the *in vitro* cytotoxicity of CCGH at different CDDP concentrations of up to 50 μg/mL (166 μM) (CDDP equivalents). We evaluated the CCGH solution with collagenase D (100 μg/mL) and without collagenase. The MKN45-Luc cells (5000 cells/well, 96-well plate) were treated with the above two CCGH solutions or free CDDP solution for 48 h, and cell viability was assessed using the Cell Count Reagent SF (Nacalai Tesque, Inc., Kyoto, Japan). The cell viability was calculated as the percentage of live cells relative to that of PBS-treated control cells. The experiment was performed in four replicate wells, and measurements were conducted three times for each well. The IC50 values of CCGH, degraded CCGH, and free CDDP were calculated by fitting a sigmoid curve using R version 3.6.3 (The R Foundation for Statistical Computing, Vienna, Austria).

### 2.8. Evaluation of In Vivo Retention of CDDP after CCGH Administration

The serum CDDP concentration was measured to evaluate the *in vivo* retention of CDDP after CCGH intraperitoneal administration. Either CCGH (10, and 30 mg/kg CDDP equivalents), free CDDP solution (4 mg/kg) or gelatin solution (equivalent to gelatin of CCGH (30 mg/kg)) was injected into the peritoneal cavities of five-week-old female ddY mice. At pre-determined time intervals (1, 3, 24, 72, and 168 h), the blood was withdrawn directly by cardiac puncture, and serum CDDP concentrations were measured with a polarized Zeeman Z-8000 atomic absorption spectrophotometer. The pharmacokinetic parameters, including the area under the serum concentration-time curve (AUC), the mean residence time (MRT), the elimination half-life (t (1/2)), the clearance (CL), and the distribution volume (Vd), were calculated for each animal by integration to infinity [41].

### 2.9. Evaluation of In Vivo Toxicity Associated with CCGH

To evaluate the systemic adverse effects, we monitored weight changes. In addition, hematological examinations were carried out to evaluate myelosuppression and nephrotoxicity. Herein, CCGH (10 and 30 mg/kg CDDP equivalents), free CDDP solution (2 and 4 mg/kg), or gelatin solution (equivalent to gelatin of CCGH (30 mg/kg)) were injected into the peritoneal cavities of five-week-old female ddY mice on day 1. On day 10, the blood was withdrawn by cardiac puncture, and the numbers of white blood cells (WBCs), red blood cells (RBCs), platelets, and the serum concentrations of blood urea nitrogen (BUN) and creatinine (Cre) were analyzed at Nihon Rinsho, Inc. (Kyoto, Japan). The mice’s body weight was measured on days 1, 3, 5, 7, and 10.

### 2.10. Evaluation of In Vivo Anticancer Efficacy of CCGH

The *in vivo* anticancer efficacy of CCGH was evaluated using a murine peritoneal dissemination model of human gastric cancer MKN45-Luc cell line. The MKN45-Luc cell suspension (5 × 10^6^ cells/mouse in 200 μL PBS) was inoculated into the peritoneal cavities of six-week-old female BALB/c nude mice on day 1. On day 4, luciferin (2 mg/mouse) dissolved in 200 μL DDW was injected into the peritoneal cavities of the mice, and macroscopic *in vivo* luminescence imaging was carried out using an *In vivo* imaging system (IVIS) (IVIS spectrum, SPI, Tokyo, Japan) for grouping based on the total counts of luminescence at the abdominal sites of the mice. The mice were assigned to the following eight groups: CCGH groups (10, 20, and 30 mg/kg CDDP equivalents); free CDDP solution groups (2, 3, and 4 mg/kg); gelatin solution group (equivalent to gelatin of CCGH (30 mg/kg)); PBS group. These were injected into the peritoneal cavities on day 5, 12, and 19. The survival rate was investigated to evaluate the *in vivo* anticancer efficacy. In addition, the body weight changes were also investigated as an indicator of the systemic side effects.

### 2.11. Statistical Analyses

All results are expressed as mean ± standard deviation. For comparisons of values between the groups at each time point, one-way analysis of variance (ANOVA) with the Tukey–Kramer post hoc test. The hematological data were compared using the Kruskal–Wallis test with the Steel post hoc test. Survival analysis was performed using the Kaplan–Meier method, and the log-rank test compared differences. *p* < 0.05 was considered statistically significant. All statistical analyses were performed with the JMP® Pro, version 16 (SAS Institute, Cary, NC, USA).

## 3. Results

### 3.1. Characterization of CCGH: In Vitro

**Figure 2:**
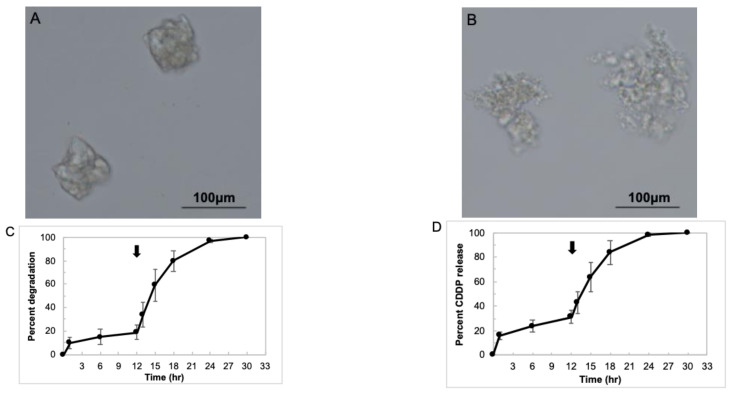
Characterization of CDDP-crosslinked gelatin hydrogels (CCGH). (**A**) A microscopic image of dried CCGH. Scale bar: 100 μm. (**B**) A microscopic image of CCGH dispersed in DDW. Scale bar: 100 μm. (**C**) Degradation profiles of CCGH. Collagenase D was added at the time indicated by the arrow (12 h). (*n* = 6). Data were represented as mean ± SD. (**D**) CDDP release profiles from CCGH. Collagenase D was added at the time indicated by the arrow (12 h). (*n* = 6). Data were represented as mean ± SD.

The microscopic images of dried CCGH and CCGH dispersed in DDW are shown in Figure 2A,B, respectively. CCGH had an irregular shape under both conditions, and the diameter was about 75–100 μm in the dry state and about 120–180 μm after DDW swelling. The CDDP content of 1 mg of CCGH was 68.4 ± 2.37 μg. Figure 2C shows the *in vitro* degradation profiles of CCGH. In PBS at 37 °C, CCGH degraded to approximately 10% in the first hour and then gradually degraded, reaching the degradation rate of approximately 20% in 12 h. After changing to PBS containing collagenase, CCGH degraded rapidly and wholly degraded in 30 h. On the other hand, the gelatin hydrogels prepared by the same method without CDDP were degraded immediately in PBS at 37 °C. Figure 2D shows the *in vitro* CDDP release profiles from CCGH. In PBS at 37 °C, the initial burst of approximately 16% was observed in 1 h. With the addition of collagenase, CDDP was released rapidly along with CCGH degradation, with almost all CDDP being released in 30 h.

### 3.2. Optimization of CCGH Preparation Conditions

**Figure 3:**
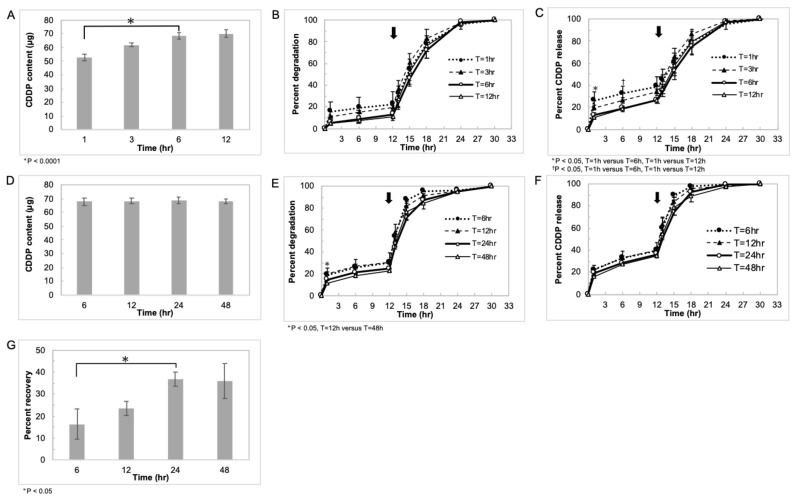
Characterization of CCGH prepared by varying the preparation conditions *in vitro*. (**A**–**C**) CDDP content per 1 mg CCGH (**A**), degradation profiles (**B**), and CDDP release profiles (**C**) of CCGH prepared by varying the stirring time of the gelatin aqueous solution and the CDDP aqueous solution at 40 °C (1 h; dotted line, 3 h; dashed line, 6 h; thick solid line, and 12 h; thin solid line) (*n* = 3). Data were represented as mean ± SD. Collagenase D was added at the time indicated by the arrow (12 h). (**D**–**G**) CDDP content per 1 mg CCGH (**D**), degradation profiles (**E**), CDDP release profiles (**F**), and recovery rate (**G**) of CCGH prepared by varying the standing time of the stirred solution at 4 °C (6 h; dotted line, 12 h; dashed line, 24 h; thick solid line, and 48 h; thin solid line). (*n* = 3). Data were represented as mean ± SD. Collagenase D was added at the time indicated by the arrow (12 h).

Figure 3A–C show the CDDP content, *in vitro* degradation profiles, and *in vitro* CDDP release profiles of CCGH prepared by varying the stirring time (1, 3, 6, and 12 h) of the gelatin aqueous solution and the CDDP aqueous solution at 40 °C, respectively. The CDDP content increased depending on the length of stirring time. Only a slight difference was shown between the stirring time of 6 h and 12 h. In the degradation and CDDP release profiles, CCGH degraded slowly, and CDDP was slowly released as the stirring time increased, but no clear difference was observed between the 6 and 12 h. Figure 3D–G show the CDDP content, *in vitro* degradation profiles, *in vitro* CDDP release profiles, and recovery rate of CCGH prepared by varying the standing time (6, 12, 24, and 48 h) at 4 °C, respectively. There were no apparent differences in the CDDP content, degradation profiles, and CDDP release profile depending on the length of the standing time. Interestingly, the longer the standing time, the better the recovery rate, but there was no significant difference between 24 h and 48 h. Based on these findings, CCGH prepared with a stirring time of 6 h and a standing time of 24 h was used for subsequent experiments.

### 3.3. Evaluation of In Vitro Cytotoxicity of CCGH

**Figure 4:**
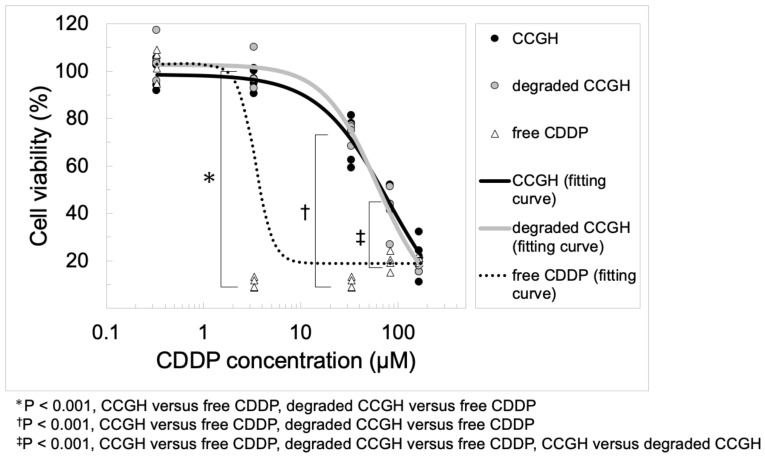
*In vitro* cytotoxicity of free CDDP solution and CCGH with and without degradation by collagenase. CCGH without degradation by collagenase (black circle), CCGH with degradation by collagenase (gray circle), and free CDDP solution (white triangle) (*n* = 4). The line means fitting curve.

Figure 4 shows the *in vitro* cytotoxicity of free CDDP solution and CCGH with and without degradation by collagenase in the MKN45-Luc cells. Both CCGH showed a decrease in cell viability depending on the CDDP concentration. However, the decrease in cell viability was mild compared to free CDDP solution, especially at low concentrations of CDDP. In CCGH, there was no difference in cytotoxicity with or without prior degradation. The IC50 values of CCGH, degraded CCGH, and free CDDP were 78.1, 61.3, and 4.29 (μM), respectively.

### 3.4. Evaluation of In Vivo Retention of CDDP after CCGH Administration

**Figure 5:**
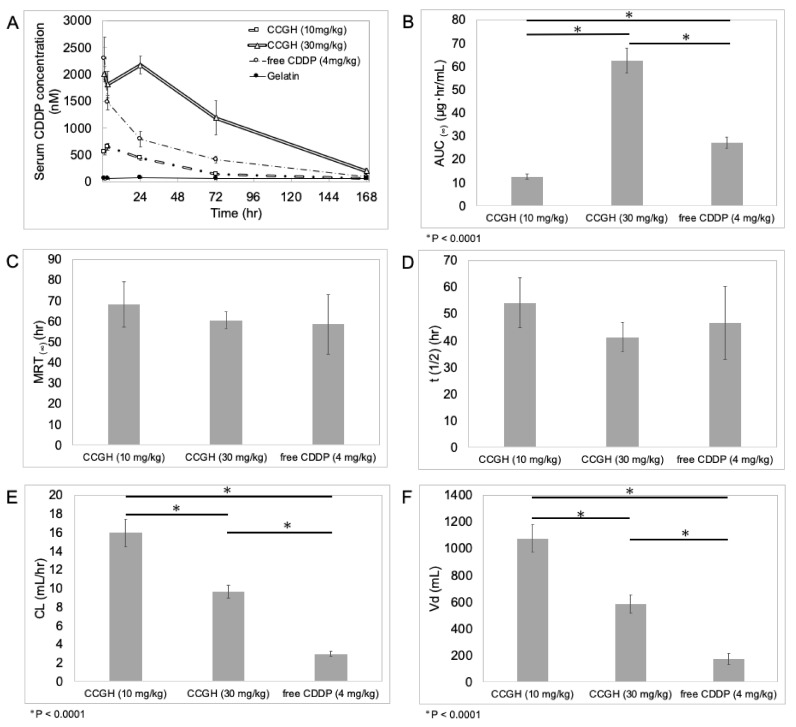
*In vivo* retention evaluation of CDDP after intraperitoneal administration. (**A**) Serum CDDP concentration profiles after intraperitoneal administration of free CDDP solution, CCGH, and gelatin solution. CCGH (10 mg/kg; double dot-dash line), CCGH (30 mg/kg; double line), free CDDP solution (4 mg/kg; dot-dash line), and gelatin solution (thin solid line). (*n* = 5). Data were represented as mean ± SD. (**B**–**F**) Main pharmacokinetic parameters of CCGH (10, 30 mg/kg) and CDDP (4 mg/kg). (*n* = 5). (**B**) AUC, area under the serum concentration–time curve; (**C**) MRT, mean residence time; (**D**) t (1/2), elimination half-life; (**E**) CL, clearance; (**F**) Vd, distribution volume.

Figure 5A shows the serum CDDP concentration profiles after intraperitoneal administration of free CDDP solution and CCGH. In the free CDDP solution group, the serum CDDP concentration was approximately 2330 nM after 1 h of administration and then decreased rapidly. On the other hand, in the CCGH (30 and 10 mg/kg) groups, the serum CDDP concentration after 1 h of administration was lower (approximately 1990 and 550 nM, respectively) despite much more CDDP (7.5 and 2.5 times, respectively), which was maintained until 24 h of administration, and the subsequent decline was gradual. The pharmacokinetic parameters of CCGH (10 and 30 mg/kg) and free CDDP (4 mg/kg) are shown in Figure 5B–F. The AUC of CCGH (30mg/kg) was increased by 2.3-fold, and the difference was significant (*p* < 0.001) compared to that of free CDDP (4 mg/kg). However, considering the dose of CDDP (7.5-fold), that of CCGH (30 mg/kg) was very low. Similarly, the AUC of CCGH (10 mg/kg) was significantly decreased by 2.1-fold compared to that of free CDDP (4 mg/kg) despite a 2.5-fold higher CDDP dose. There was not a significant difference of the MRT and the t (1/2) between CCGH and free CDDP. The CL and Vd of CCGH (10 and 30 mg/kg) were significantly increased compared to those of free CDDP (4 mg/kg).

### 3.5. Evaluation of In Vivo Toxicity of CCGH

**Figure 6:**
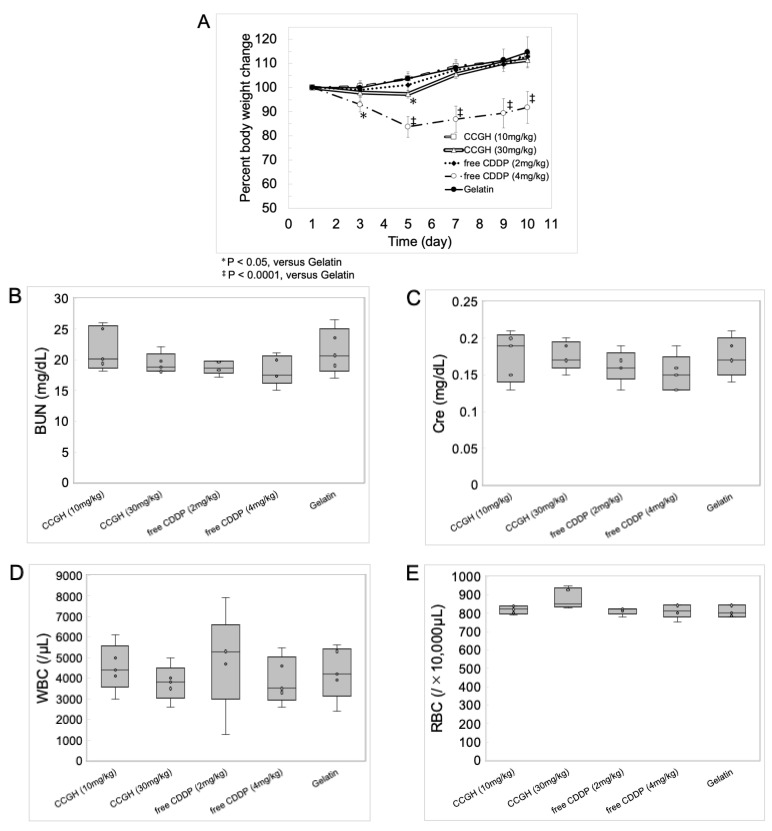

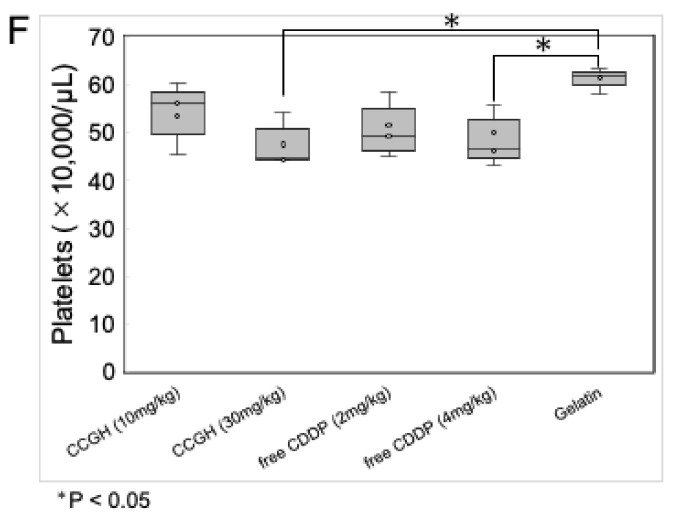
*In vivo* toxicity evaluation after treatment (day 1) in mice. (**A**) The profiles of body weight change. CCGH (10 mg/kg; double dot-dash line), CCGH (30 mg/kg; double line), free CDDP solution (2 mg/kg; dotted line), free CDDP solution (4 mg/kg; dot-dash line), and gelatin solution (thin solid line). (*n* = 5). Data were represented as mean ± SD. (**B**–**F**) Hematological examination data on day 10. (**B**) Serum concentrations of blood urea nitrogen (BUN). (**C**) Serum concentrations of creatinine (Cre). (**D**) Numbers of white blood cells (WBCs). (**E**) Numbers of red blood cells (RBCs). (**F**) Numbers of platelets. CCGH (10 mg/kg), CCGH (30 mg/kg), free CDDP solution (2 mg/kg), free CDDP solution (4 mg/kg), and gelatin solution. (*n* = 5).

Figure 6A shows the profiles of body weight change after intraperitoneal administration of free CDDP solution, CCGH, and gelatin solution. The dose-dependent weight loss was observed after administration in the free CDDP solution groups. The weight loss in the CCGH (30 mg/kg) group was observed, but it was mild compared to the free CDDP solution groups despite high CDDP doses. No apparent weight loss was observed in the CCGH (10 mg/kg) group. On the other hand, there were no significant differences in the serum BUN (Figure 6B) and Cre (Figure 6C) concentrations and the number of WBCs (Figure 6D) and RBCs (Figure 6E) in the blood between the CCGH, the free CDDP solution, and gelatin solution groups. In CCGH (30 mg/kg) and free CDDP (4 mg/kg) groups, there was significant differences in the number of platelets (Figure 6F) in the blood compared to gelatin group, though the decrease was negligible.

### 3.6. Evaluation of In Vivo Anticancer Efficacy of CCGH

**Figure 7:**
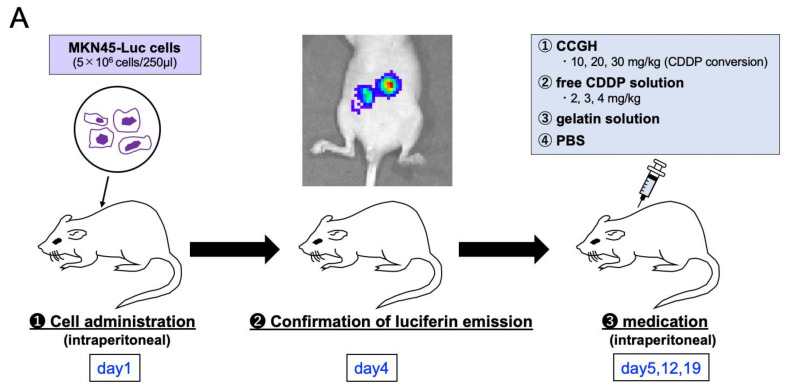

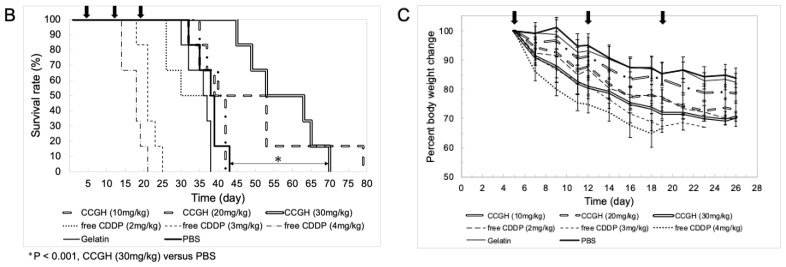
*In vivo* anticancer efficacy and toxicity evaluation after treatment in a mouse model with peritoneal metastases. (**A**) A schema of animal experiments. (**B**) Survival time profiles. CCGH (10 mg/kg; double dot-dash line), CCGH (20 mg/kg; double dashed line), CCGH (30 mg/kg; double line), free CDDP solution (2 mg/kg; dotted line), free CDDP solution (3 mg/kg; dashed line), free CDDP solution (4 mg/kg; dot-dash line), gelatin solution (thin solid line), and PBS (thick solid line). (*n* = 6). Drugs were injected at the time indicated by arrows (day 5, 12, and 19). (**C**) Body weight change profiles from the first injection (day 5) up to 7 days after the third injection (day 26). CCGH (10 mg/kg; double dot-dash line), CCGH (20 mg/kg; double dashed line), CCGH (30 mg/kg; double line), free CDDP solution (2 mg/kg; dotted line), free CDDP solution (3 mg/kg; dashed line), free CDDP solution (4 mg/kg; dot-dash line), gelatin solution (thin solid line), and PBS (thick solid line). (*n* = 6). Drugs were injected at the time indicated by arrows (day 5, 12, and 19).

Figure 7A shows a scheme of animal experiments. Figure 7B shows the survival time profile after treatment in a mouse model of peritoneal metastases. The survival rate in the high dose of CCGH (30 mg/kg) treatment group was significantly higher than that in the PBS group (*p* < 0.001). In the free CDDP solution groups, all mice administered a high dose (4 mg/kg), and one mouse administered a medium dose (3 mg/kg) died before the third injection. The remaining five mice in the medium dose died within six days after the third injection because of chemotoxicity. In the medium and low-dose CCGH treatment groups (20 and 10 mg/kg) and the low-dose free CDDP group (2 mg/kg), survival time was not significantly prolonged compared to the control groups.

Figure 7C shows the profile of body weight change from the first injection (day 5) to 7 days after the third injection (day 26). These profiles were similar to non-tumor-bearing mice (Figure 6). In the CCGH treatment groups, the dose-dependent weight loss after injection was more evident than in the control groups.

## 4. Discussion

We have previously reported that CDDP-incorporated gelatin hydrogels for CDDP controlled release with highly efficient anticancer efficacy with negligible side effects [37,38,39,40]. The hydrogels were prepared by a two-step method, wherein gelatin was crosslinked with glutaraldehyde or dehydrothermal treatment, and CDDP was incorporated. Glutaraldehyde is a widely-used chemical crosslinking agent that forms a strong crosslinked gelatin network. Still, it has significant cytotoxicity and is a biohazard problem, limiting its application in commercial products [42]. On the other hand, the dehydrothermal treatment results in crosslink formation through a water condensation reaction. This reaction occurs under long-term (usually several days) high temperature and vacuum conditions (T > 100 °C, *p* < 100 mTorr) [43]. In addition to glutaraldehyde and dehydrothermal treatments, a number of small molecule crosslinking agents such as epoxy compounds [44], carbodiimide compounds [43], genipin [45], citric acid [46,47], isocyanate [44], enzymes such as transglutaminase [48], high-energy electron beams [49,50], gamma rays [51], UV [52], plasma [53], and visible light [54], have been extensively employed for crosslinking gelatin. Furthermore, some chemical modifications of gelatin to introduce crosslinking functionalities of gelatin methacryloyl, are also reported [55]. Nonetheless, some exhibit toxicity and require special equipment and complicated procedures.

In this study, we developed a novel preparation method for CCGH where CDDP was used as a crosslinking agent for gelatin. It is a one-step method of simple mixing CDDP and gelatin, resulting in gelatin crosslinking and CDDP incorporation. It does not require small molecule crosslinking agents, enzymes, or instruments for gelatin crosslinking. The procedure is simple and easy, and the preparation time is short.

CDDP is a metal coordination compound that consists of square-planar platinum (II) center coordinated to two ammonia ligands and two chloride ligands with a cis-ligand conformation. Even though the ammonia ligands are relatively inert, the chloride ligands are relatively labile and prone to nucleophilic substitution [56]. It is demonstrated that the binding mechanism between CDDP and gelatin molecules involves a coordinate bond between platinum atom and carboxyl groups of gelatin molecules using Fourier-transform infrared (FT-IR) spectra [57], although CDDP was added to the gelatin nanoparticle which had been already crosslinked with glutaraldehyde in their study. The ligand exchange of platinum from chloride to the carboxyl groups of gelatin occurs quickly in an aqueous solution. Since CDDP has two chloride ligands, CDDP itself can likely act as a crosslinking agent for gelatin in the present method. However, the bond between CDDP and gelatin is considered reversible due to its low nucleophilicity. Therefore, it was confirmed that CCGH prepared by mixing CDDP and gelatin was sufficiently crosslinked and showed a CDDP sustained release. The *in vitro* degradation test (Figure 2C) indicates that CCGH was sufficiently crosslinked and not degraded, although 10% of gelatin was degraded initially. On the other hand, the gelatin hydrogels prepared by the same method without CDDP were degraded immediately in PBS at 37 °C. There was the difference in the degradation profiles between the gelatin hydrogels with and without CDDP. We believe that the finding is due to the crosslinking of gelatin molecules with CDDP. Based on these findings, we can say with certainty that gelatin is crosslinked with CDDP via a coordinate bond between the platinum atom and the carboxyl groups of gelatin molecules through the exchange in the two ligands of platinum. Moreover, the *in vitro* CDDP release test (Figure 2D) indicates that CCGH released CDDP continuously with an initial burst of approximately 16%. It is demonstrated that CDDP incorporated into gelatin was released from the gelatin hydrogel when the hydrogel was degraded to generate water-soluble gelatin fragments [37,57]. Based on this concept, we can confidently say that CDDP release from CCGH was based on the degradation of CCGH. It was investigated how the difference in preparation conditions, specifically the stirring time of gelatin aqueous solution and CDDP aqueous solution at 40 °C, and the standing time of mixed solution after stirring at 4 °C, affects the CDDP content and the degree of crosslinking. The CDDP content and the degree of crosslinking tended to increase depending on the stirring time at 40 °C (Figure 3A,B). However, the difference between 6 h and 12 h was small. On the other hand, the standing time at 4 °C did not affect the CDDP content and the degree of crosslinking (Fig. 3D and 3E). The CDDP release test showed a similar trend (Figure 3C,F). Based on these findings, we think the mechanism of gelatin crosslinking with CDDP as follows. The binding of CDDP and gelatin occurs during the stirring at 40 °C because the molecules collision frequency of CDDP and gelatin molecules increases due to a promoted molecular motion of gelatin. However, most of the binding is likely formed by only one ligand exchange. Therefore, the hydrogel is not completely formed only by stirring at 40 °C yet. The subsequent cooling process at 4 °C causes the intact property of gelatin itself to form a physically bound hydrogel, bringing CDDP and gelatin closer together. As a result, another ligand exchange is triggered, and the gelatin crosslinking with CDDP is completed to form the hydrogel. A short cooling time (no longer than 6 h) at 4 °C may suffice for another ligand exchange. However, considering the result that a short cooling time at 4 °C causes a reduced recovery rate (Figure 3G), a short cooling time may cause inadequate physical hydrogel formation of gelatin itself, resulting in less yield during the crushing procedure with sieves. These are just the possible idea. Further study needs to experimentally confirm this statement. In addition, these findings indicate that the degree of crosslinking and CDDP release was controlled by adjusting the stirring time at 40 °C, although almost all CDDP molecules probably bind to gelatin at 40 °C for 6–12 h. It is necessary to modify the preparation conditions, such as increasing the amount of charged CDDP, prolonging the reaction temperature, and changing the concentration and molecular weight of gelatin, aiming at further increase in the degree of crosslinking.

The molecular mechanism of CDDP anticancer activity involves the transport of CDDP into the tumor cells through copper transporter 1 (CTR1), followed by chloride ligand(s) replacement by water molecules, resulting in the formation of cationic hydrate, such as cis-[Pt(NH3)2Cl(OH2)]+ and cis-[Pt(NH3)2(OH2)2]2+ [4,5]. This replacement is caused by the chloride ion concentration gradient between the extracellular matrix (about 100 mM) and the cytoplasm (about 4 mM). These cationic hydrates are highly reactive and bind to DNA (mainly the nitrogen atom at position 7 of the guanine base) in the nucleus to form intra and inter-stranded crosslinks, changing DNA structure and causing DNA damage. This DNA damage may cause cell cycle arrest and apoptosis in rapidly proliferating tumor cells. In addition, other anticancer mechanisms of CDDP include the acidification of the cytoplasm, endoplasmic reticulum stress, disruption of RNA transcription, inhibition of key carcinogenic proteins, decrease in metabolic plasticity of cancer cells, and changes in their mechanobiology [5,58,59,60]. As mentioned above, it has been demonstrated that the binding mechanism between CDDP and gelatin in CCGH involves a coordinate bond through the ligand exchange of platinum from chloride to the carboxyl groups of gelatin. Based on this, CDDP released from CCGH due to CCGH degradation by collagenase is actually considered to be free CDDP or CDDP in which one or two chlorides are replaced with gelatin fragments. The *in vitro* cytotoxicity test (Figure 4) demonstrated that CDDP released from the CCGH did show anticancer activity, although it was lower than free CDDP. Although the undegraded CCGH treatment showed an anticancer activity, most undegraded CCGH may be degraded by matrix metalloproteinase (MMP) [38] secreted from the cancer cells during the 48 h incubation. CDDP released from the CCGH probably has anticancer activity mechanisms similar to free CDDP, but the detailed mechanism needs further investigation.

The pharmacokinetic parameters (Figure 5B–F) were assessed from the serum CDDP concentration profiles after intraperitoneal administration (Figure 5A). There was no significant difference in the MRT and the t (1/2) between CCGH and free CDDP solution. These results indicate that most of the CDDP in the blood after intraperitoneal administration of CCGH may be a state close to the free form of CDDP or CDDP chelated with small degradation fragments of gelatins. In other words, CDDP released from CCGH may remain in the abdominal cavity without transferring into the blood if not sufficient degradation. Despite the higher net CDDP dosage in CCGH injected groups, the CL of CCGH injected groups was significantly high compared to that of free CDDP injected group. This observation also supported that CDDP in CCGH complex would be difficult to absorb into bloodstream. In addition, due to AUC value of CCGH injected groups derived from less absorption in bloodstream, it was major influence on the high CL and Vd value compared with those of a CDDP injected group. Taken together, these results suggest that CDDP is gradually released from CCGH complex in the abdominal cavity by degradation of gelatin. This expected pharmacokinetics of CDDP may help to reduce the side effects. Following the intraperitoneal administration of CCGH, the body weight loss was minimal despite higher CDDP doses compared to free CDDP solution (Figure 6A and Figure 7C). Interestingly, the weight loss was less, even though the administration of CCGH (30 mg/kg) maintained a high serum CDDP concentration for extended periods than that of free CDDP solution (4 mg/kg). It is conceivable that CDDP chelated with small degradation fragments of gelatin circulating in the blood is difficult to be taken up by normal cells.

The MMP secreted by cancer cells assists in the degradation of CCGH to release CDDP near the cancer site [38]. In addition, it has also been reported that CDDP’s anticancer activity depends on the dose and exposure time [61]. Therefore, the local administration of CCGH, which is locally retained for a long time and releases CDDP slowly, may not only reduce adverse effects but also show better anticancer efficacy. The anticancer efficacy of intraperitoneal administration of CCGH was investigated using a murine gastric cancer peritoneal dissemination model (Figure 7A). It has been reported that the maximum tolerated dose of free CDDP solution in mice ranges from 5 to 10 mg/kg [62,63]. In this study, all mice injected with free CDDP (4 mg/kg) solution and one mouse injected with free CDDP (3 mg/kg) solution died before the third injection, while all the remaining five mice injected with free CDDP (3 mg/kg) solution died within six days after three times injections because of chemotoxicity. The free CDDP (2 mg/kg) solution group tolerated injections three times but showed no prolongation of survival compared to the survival rate in the control groups. On the other hand, the CCGH (30 mg/kg) group showed a significantly prolonged survival time (Figure 7B). In addition, the degree of weight loss after injection was mild in the CCGH groups compared with the weight loss in the free CDDP groups, even though the CDDP dose was much higher (Figure 7C). These results indicate that the local sustained release of CDDP from CCGH shows a better anticancer efficacy while reducing the side effects.

To date, a variety of carrier materials have been developed for sustained release of CDDP, such as collagen/gelatin [39,64], alginic acid [65], hyaluronic acid [66], chitosan [67], dextran [68], polylactic acid (PLA) [69], poly(L-glutamic acid) (PGA) [70], PLA- or PGA-related copolymers [71,72], ceramics [73,74], and lipids [75]. For some systems, the CDDP release from materials is regulated by a simple drug diffusion through the material. Therefore, it is technically difficult to predict the time profile of CDDP sustained release. On the other hand, there is a report on another strategy where the drug is released in response to MMP [76]. For the present system of gelatin hydrogels, the CDDP release can be controlled only as a result of the degradation of carrier material gelatin. Based on this, it is easy to predict the time profile of CDDP release. In addition, the carrier material is completely degraded after the complete release of CDDP. The inflammation induced by the residual material is not a problem due to gelatin’s high biocompatibility and biodegradability.

There are other platinum-based anticancer drugs, such as carboplatin and oxaliplatin. Considering their chemical structure, they may also be able to act as the crosslinking agent of gelatin. Further investigation for potential sustained release gelatin formula using other platinum-based drugs is awaited.

This study has several limitations. First, the sustained release of CDDP from the CCGH was not directly confirmed *in vivo*. As far as our knowledge, accurately measuring CDDP concentration following locally sustained release is impossible. Instead, we indirectly confirm the sustained release of CDDP by assessing the serum CDDP concentration after intraperitoneal administration of CCGH. Second, the sustained release pattern of CDDP from CCGH is not optimized. As abovementioned, it is considered that the sustained release pattern can be controlled by varying the preparation conditions such as the stirring time of gelatin aqueous solution and CDDP aqueous solution at 40 ℃, the temperature when stirring, the amount of charged CDDP, and the concentration and molecular weight of gelatin. It is desirable to investigate the *in vivo* anticancer activity and the *in vivo* toxicity of CCGH with different sustained release patterns. Third, in this study, only one cell line (MKN45-Luc) is selected for evaluating the anticancer efficacy. Therefore, CCGH requires validation in other types of cancer.

## 5. Conclusions

We developed a new preparation method of gelatin hydrogels incorporating CDDP, an anticancer drug for sustained release. By simply mixing CDDP and gelatin, CDDP functioned as a crosslinking agent of gelatin to form the gelatin hydrogel and was simultaneously incorporated into the hydrogel. Therefore, any chemical crosslinking agents, enzymes, or particular instruments for gelatin crosslinking are not required, and the preparation time is very short. The developed CCGH showed an *in vivo* retention property. The local sustained release of CDDP from CCGH *in vivo* resulted in prolonged survival with reduced toxicity in a murine gastric cancer peritoneal dissemination model. We consider that the local administration of CCGH, which allows long-term continuity because of the superior anticancer efficacy and minimal side effects than the free CDDP solution, may be an ideal treatment option for improving the survival in patients, especially in advanced-stage patients with poor chemotolerance. Further studies are required to evaluate the optimal sustained release pattern, the amount of administration, the number of doses, dosing intervals, and appropriate combinations with other anticancer drugs for its clinical application.

## Data Availability

Not applicable.
